# Genetic analysis of zebrafish homologs of human FOXQ1, *foxq1a* and *foxq1b*, in innate immune cell development and bacterial host response

**DOI:** 10.1371/journal.pone.0194207

**Published:** 2018-03-13

**Authors:** Alison M. Earley, Cameron T. Dixon, Celia E. Shiau

**Affiliations:** Department of Biology, University of North Carolina at Chapel Hill, Chapel Hill, NC, United States of America; INRA, FRANCE

## Abstract

FOXQ1 is a member of the forkhead-box transcription factor family that has important functions in development, cancer, aging, and many cellular processes. The role of FOXQ1 in cancer biology has raised intense interest, yet much remains poorly understood. We investigated the possible function of the two zebrafish orthologs (*foxq1a* and *foxq1b*) of human FOXQ1 in innate immune cell development and function. We employed CRISPR-Cas9 targeted mutagenesis to create null mutations of *foxq1a* and *foxq1b* in zebrafish. Using a combination of molecular, cellular, and embryological approaches, we characterized single and double *foxq1a* ^*bcz11*^ and *foxq1b* ^*bcz18*^ mutants. This study provides the first genetic mutant analyses of zebrafish *foxq1a* and *foxq1b*. Interestingly, we found that *foxq1a*, but not *foxq1b*, was transcriptionally regulated during a bacterial response, while the expression of *foxq1a* was detected in sorted macrophages and upregulated in *foxq1a*-deficient mutants. However, the transcriptional response to *E*. *coli* challenge of *foxq1a* and *foxq1b* mutants was not significantly different from that of their wildtype control siblings. Our data shows that *foxq1a* may have a role in modulating bacterial response, while both *foxq1a* and *foxq1b* are not required for the development of macrophages, neutrophils, and microglia. Considering the implicated role of FOXQ1 in a vast number of cancers and biological processes, the *foxq1a* and *foxq1b* null mutants from this study provide useful genetic models to further investigate FOXQ1 functions.

## Introduction

FOXQ1 is a member of the forkhead-box (FOX) transcription factor gene family, which contains a winged helix DNA binding domain and has important functions in development, cancer, aging, cell cycle regulation, cell migration, and other diverse cellular processes [1–5]. Previous studies in mammals show that the functions of FOXQ1 include promoting epithelial differentiation [6–11], inhibiting smooth muscle differentiation [12], mediating hair development [13,14], controlling gastric acid production and secretion in stomach mucous cells [15,16], regulating glucose metabolism [17], as well as possible immunological functions [18,19]. Additionally, upregulation of FOXQ1 is found in a large host of cancer types, possibly to regulate cell proliferation and invasion, including breast [7,20,21], colorectal [22–25], pancreatic [26,27], gastric [28–31], bladder [32], liver [33–36], lung [37,38], ovarian [39,40], and glioma [41]. With these findings, FOXQ1 has generated much interest into its functions and mechanisms.

In light of the interactions of innate immune cells, particularly macrophages, in tumor development, progression, and metastasis [42–48], and possible roles of FOXQ1 in regulating these processes [49,50], we generated gene knockouts of the two zebrafish orthologs (*foxq1a* and *foxq1b*) of human FOXQ1 using CRIPSR-Cas9 targeted mutagenesis. Using single and double *foxq1a* and *foxq1b* mutants, we investigated whether FOXQ1 is critical for the development and function of macrophages and other innate immune cells. Establishing a function for FOXQ1 in innate immune regulation can provide new understanding of its role in various cancers.

This study provides the first genetic mutant analyses of zebrafish *foxq1a* and *foxq1b*. We found RNA expression of both copies of *foxq1* in the developing craniofacial structures as previously reported [51,52], in addition to *foxq1a* expression in sorted macrophages as well as in the yolk region. Neither single nor double *foxq1a* and *foxq1b* mutants had overt gross morphological defects or notable deficiency in the development of macrophages, neutrophils, and microglia. Furthermore, *foxq1a* and *foxq1b* mutants exhibited a typical transcriptional response to bacterial challenge, but *foxq1a* is interestingly downregulated during this response in wildtype. Considering the implicated role of FOXQ1 in a vast number of cancers and biological processes, the *foxq1a* and *foxq1b* mutants generated in this study provide new genetic models to further dissect the functions of FOXQ1.

## Results

### Generation of *foxq1a* ^*bcz11*^ and *foxq1b* ^*bcz18*^ null alleles using CRISPR-Cas9

**Fig 1:**
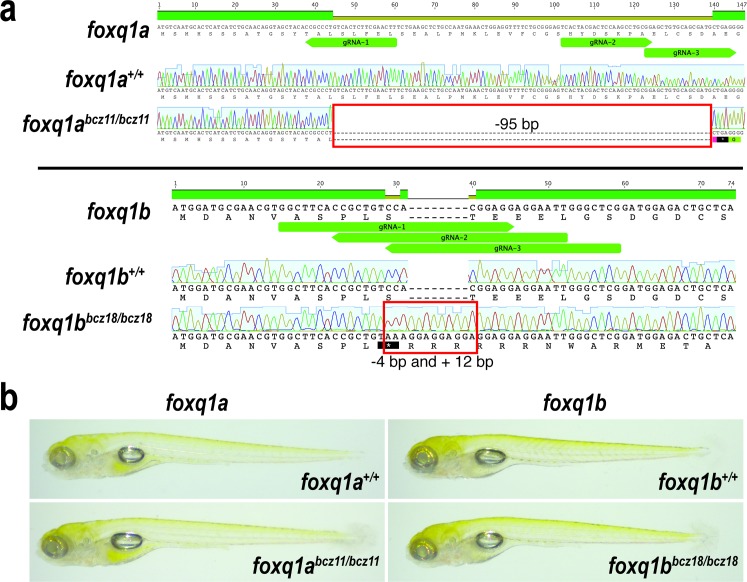
Sequence analysis of *foxq1a*^*bcz11*^ and *foxq1b*^*bcz18*^ mutations generated by CRISPR-Cas9 targeting. **a** Top, DNA and amino acid sequences of the 5’ end of *foxq1a* coding region. DNA sequencing chromatograms show the expected foxq1a sequence in the wildtype sibling, and a large 95 base pair deletion (red box) in the homozygous *bcz11* mutant, which causes a premature stop (black box with an asterisk) at the beginning of the gene. Bottom, DNA and amino acid sequences of the 5’ end of *foxq1b* coding region. DNA chromatograms show wildtype sequence in *foxq1b* wildtype sibling, while the homozygous *bcz18* mutant carries a 4 bp deletion and 12 bp insertion (red box) causing a nonsense mutation at the beginning of the gene. gRNA, target sites of the guide RNAs used. **b** Whole mount live imaging of 5 dpf larvae show normal gross morphological development of *foxq1a*^*bcz11*^ and *foxq1b*^*bcz18*^ mutants as compared to wildtype siblings (at least 10 animals were analyzed per genotype group).

We employed the highly effective CRISPR-Cas9 mediated targeted mutagenesis in zebrafish as previously described [53,54] to create loss-of-function mutations at the beginning of *foxq1a* and *foxq1b* genes. We isolated two mutant alleles of interest: *foxq1a*^*bcz11*^ and *foxq1b*^*bcz18*^ (Fig 1A). These are frameshift mutations that lead to a premature stop codon at the beginning of each respective gene: *bcz11* has a large 95 base pair (bp) deletion in *foxq1a* that is immediately followed by a premature stop codon, and *bcz18* has a 4 bp deletion and a 12 bp insertion in *foxq1b* that causes a premature stop codon within the start of the indel (Fig 1A). RT-PCR and sequencing analysis confirmed expression of the expected nonsense transcripts from the *bcz11* and *bcz18* mutations, and no alternative splicing was found (S1 Fig). The data indicate that *bcz11* and *bcz18* represent null alleles of their respective genes by producing nonsense mRNAs that would prevent Foxq1a and Foxq1b protein expression, respectively. For each gene target, three guide RNAs (gRNAs) were used in combination to increase the efficiency of the mutagenesis and the probability of generating a large indel mutation (Fig 1A). At 5 days post-fertilization (dpf) when zebrafish have well established organ systems and are free-swimming larvae, we examined the gross morphology of the homozygous mutants (Fig 1B). We found that both *foxq1a*^*bcz11*^ and *foxq1b*^*bcz18*^ homozygous mutants were indistinguishable from wildtype siblings (Fig 1B), displaying normal-sized inflated swim bladders, and grossly normal head and body structures that were no different from wildtype. However, we cannot exclude the possibility that craniofacial and specific organ phenotypes may be present in the mutants without further detailed analysis, in regions that normally express *foxq1a* and *foxq1b* [51,52]. The viability of *bcz11* and *bcz18* mutants, at least up to larval stages, appeared to be normal, as we consistently found ~25% of the progenies to be mutants from a heterozygous incross.

### Characterization of *foxq1a* and *foxq1b* gene expressions

**Fig 2:**
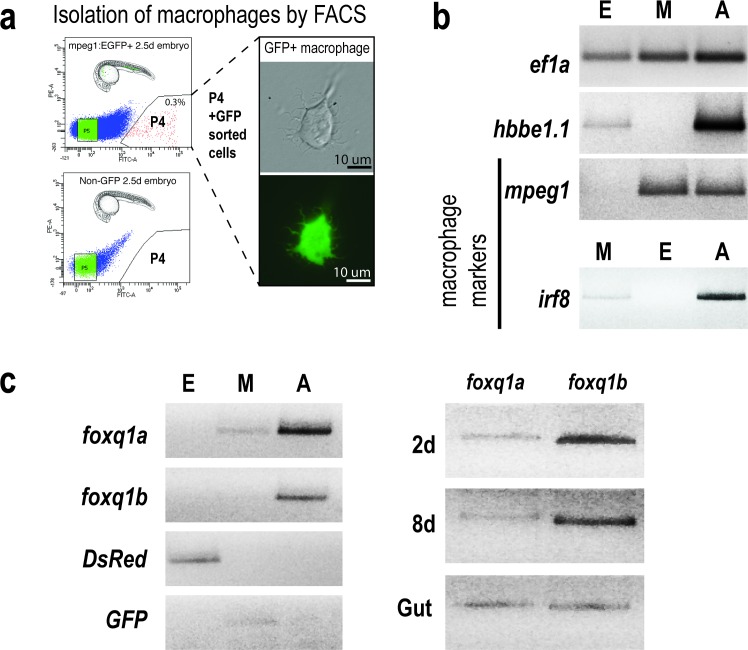
Gene expression analysis of *foxq1a* and *foxq1b*. **a** Isolation of macrophages based on GFP expression by FACS in *mpeg1*:*EGFP* transgenic zebrafish embryos at 2.5 dpf. Non-fluorescent embryos were used as a negative control for gating. Top left, P4 shows the cell fraction sorted as GFP+ macrophages, also shown in the right panels in brightfield and green channel. **b** RT-PCR analysis of gene markers validated the different cell populations sorted by FACS, as denoted by E, erythrocytes; M, macrophages; A, all remaining non-fluorescent cells. The following genes were used: translation elongation factor 1 (*ef1a*) as a reference marker, hemoglobin beta embryonic-1.1 (*hbbe1*.*1*) as an erythrocyte marker, and macrophage expressed gene 1 (*mpeg1*) and interferon regulatory factor 8 (*irf8*) as well-established macrophage markers in zebrafish. As expected, the ‘A’ cells expressed all genes, while erythrocytes ‘E’ cells expressed *hbbe1*.*1*, *ef1a*, and *DsRed* genes and macrophage ‘M’ cells expressed macrophage markers, *GFP*, and *ef1a*. **c** Using the sorted cell populations, we found expression of *foxq1a* in macrophages and neither gene in erythrocytes. Both genes are expressed at 2 to 8 dpf of development as well as in the adult gut tissue.

The expression patterns of *foxq1a* and *foxq1b* in zebrafish remain largely unanalyzed. Only two studies to date have examined their expressions in specific tissues of interest; one showed *foxq1b* expression in the developing jaws at 2 dpf [51] and the other reported that *foxq1a* may be expressed in the cranial suture in juvenile zebrafish (6 weeks post-fertilization) [52]. To better define the possible functions of these genes in zebrafish, we expanded the characterization of their expressions to various stages of development from 1 to 8 dpf, and tested whether they were expressed in macrophages (Fig 2). Using fluorescence-activated cell sorting (FACS), we isolated macrophages (M) based on GFP expression from 2.5 dpf zebrafish embryos carrying the macrophage-specific transgene *mpeg1*:*EGFP* (Fig 2A). To assess their expression in other cell types as additional references, we sorted erythrocytes (E) based on DsRed expression from erythrocyte-specific *gata1*:*DsRed* transgenic embryos as well as the remaining non-fluorescent cells (A) (Fig 2B and 2C). We verified the identity of the sorted cell populations by RT-PCR analysis using known markers and fluorescent reporter genes for the E, M, and A cells (Fig 2B and 2C). The data shows that while both genes are expressed from 2 to 8 dpf (Fig 2C), only *foxq1a* is expressed by macrophages and neither gene is expressed by erythrocytes (Fig 2C). Using RNA *in situ* hybridization, we found expression of *foxq1a* in the cranial region at 1 dpf that persists in the jaw at 2.5 dpf when it is also found in the body tissue along the yolk, and on the yolk (S2 Fig). By contrast, *foxq1b* was primarily found to be expressed in the ventral jaw region at 2.5 dpf (S2 Fig). While *foxq1a* RNA expression was found in macrophages isolated from wildtype embryos (Fig 2C), its level may be too low to be detectable by whole mount RNA in situ hybridization as we were unable to visualize its expression in macrophages by *in situ* (S2 Fig). Because FOXQ1 is known to be highly expressed in the mammalian gastrointestinal tract [15,16,55,56], we examined whether *foxq1a* and *foxq1b* are expressed in the adult zebrafish gut, and indeed found them both to be expressed (Fig 2C).

### Single and double *foxq1a*^*bcz11*^ and *foxq1b*^*bcz18*^ mutants show normal macrophage, neutrophil, and microglia development

**Fig 3:**
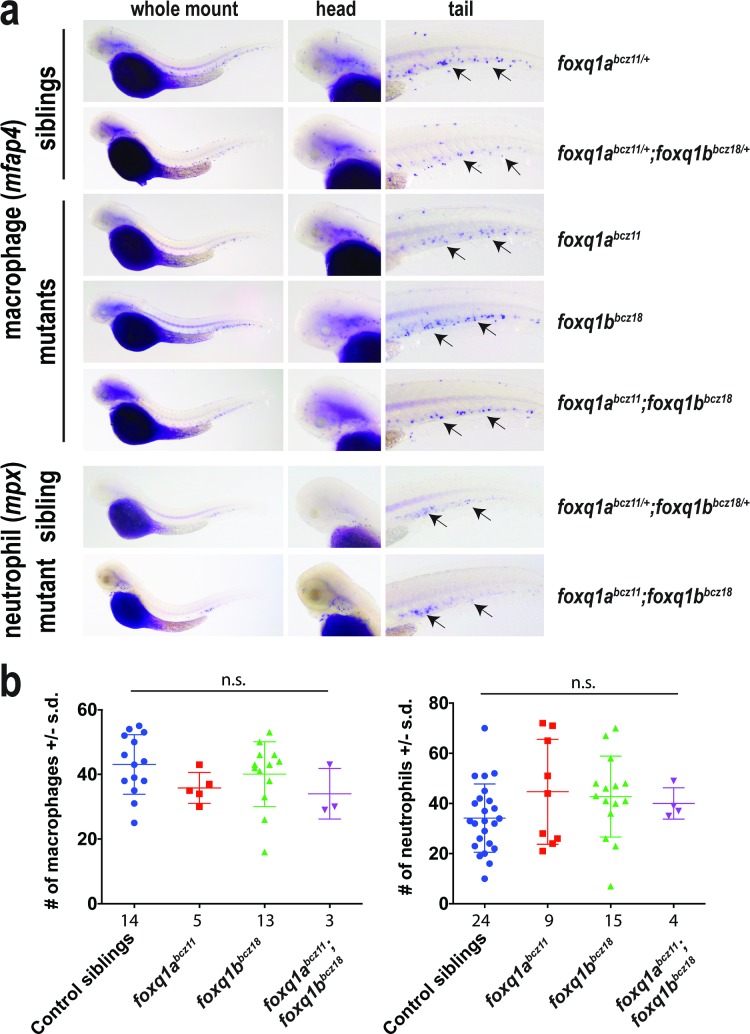
Single and double *foxq1a*^*bcz11*^ and *foxq1b*^*bcz18*^ mutants have normal macrophage and neutrophil development. **a** Whole mount *in situ* hybridization of 2.5 dpf single and double *foxq1a*^*bcz11*^ and *foxq1b*^*bcz18*^ mutants and their siblings using RNA probes for *mfap4* (macrophage marker) and *mpx* (neutrophil marker). Arrows, macrophages or neutrophils in the caudal hematopoietic tissue in the embryo tail. **b** Scatter plots showing number of macrophages and neutrophils in the tail region for each embryo quantified in the different genotype categories. n, number of embryos analyzed beneath each scatter plot. Plots report average ± standard deviation (s.d.). Multiple unpaired t-tests comparing between control siblings and each of the mutants groups were conducted with correction for multiple comparisons using the Sidak-Bonferroni method and without assuming equal variance to determine statistical significance. n.s., no statistical significance as defined by p > 0.05 was found in all the comparisons.

**Fig 4:**
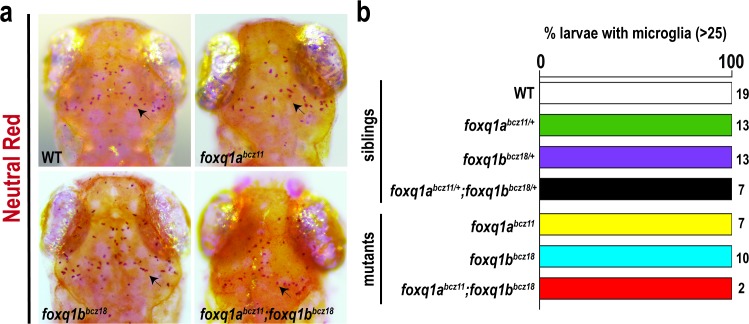
Neutral red analysis shows normal microglia development in single and double *foxq1a* and f*oxq1b* mutants at 4 dpf. **a** Neutral red staining in 4 dpf wildtype (WT) sibling, *foxq1a*^*bcz11*^, *foxq1b*^*bcz18*^, and *foxq1a*^*bcz11*^*;foxq1b*^*bcz18*^ mutants shows a stereotypical pattern of microglia in the midbrain (arrow). **b** Bar graph shows all larvae analyzed in all genotype groups had normal numbers of microglia (>25). Number of larvae analyzed (n) shown to the right of the corresponding bar.

In light of the finding that *foxq1a* is expressed in macrophages and the implicated mammalian FOXQ1 function in tumor biology that may involve innate immune cells [19], we asked whether the development of macrophages and neutrophils may be altered in the zebrafish *foxq1* mutants. By whole mount RNA in situ hybridization using a macrophage marker (*mfap4*) [57,58] and neutrophil marker (*mpx*) [59,60], we found no apparent difference in formation or distribution of these innate immune cells during development at 2.5 dpf in *foxq1a*^*bcz11*^ mutants and *foxq1b*^*bcz18*^ mutants (Fig 3A). In case of redundant or compensatory functions of *foxq1a* and *foxq1b*, we examined the double *foxq1a*^*bcz11*^*;foxq1b*^*bcz18*^ homozygous mutants, but also found no marked differences compared to wildtype siblings (Fig 3A). Quantification of macrophage and neutrophil numbers in the tail region further substantiates our finding that the mutants are not significantly different from siblings in innate immune cell development (Fig 3B). Since it is possible to have defects in macrophages at later time points, we evaluated the development of microglia, an important derivative of embryonic macrophages. We used a vital neutral red stain assay that has been shown to specifically label microglia in the larval zebrafish brain [61–63] as cells with dark red aggregates (Fig 4A). We found that all *foxq1a*^*bcz11*^, *foxq1b*^*bcz18*^ and *foxq1a*^*bcz11*^*;foxq1b*^*bcz18*^ mutants exhibited the typical pattern (Fig 4A) and numbers (Fig 4B) of microglia in the larval midbrain as those in wildtype and heterozygous control siblings and previously described for wildtype [61–65]. Taken together, the data indicates that *foxq1a* and *foxq1b* are not required for the proper development of macrophages, neutrophils, and microglia.

### *foxq1a* and *foxq1b* null mutants exhibit typical transcriptional response to *Escherichia coli* brain challenge

**Fig 5:**
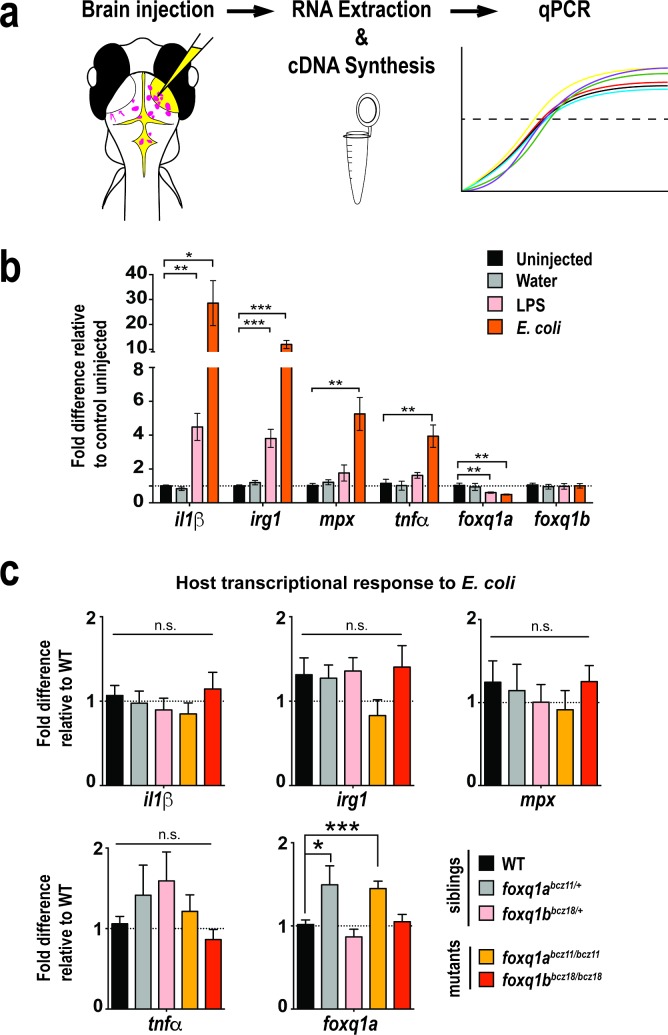
*foxq1a*^*bcz11*^ and *foxq1b*^*bcz18*^ mutants exhibit wildtype transcriptional response to *E*. *coli* challenge in the larval brain. **a** Graphical representation of the brain challenge assay. *E*. *coli* was injected into right brain tectum, and at 6 hours post injection (hpi), injected larvae were individually processed for RNA extraction, genotyping, cDNA synthesis, and qPCR analysis. **b** Bar chart shows significant upregulation of immune activation genes *il1β*, *irg1*, *mpx*, and *tnfα* after bacteria challenge at 6 hpi in wildtype larvae compared with uninjected controls. Water injected wildtype larvae exhibit no induction of activation genes, whereas LPS injected wildtype animals have a significant upregulation of *il1β* and *irg1*. After microbial activation by *E*. *coli* or LPS, a significant downregulation of *foxq1a* was found by about a factor of 2. No significant change was found for *foxq1b* after immune challenge. Data from wildtype injections validate the efficacy of the assay to activate the innate immune system. Dotted line marks no fold difference at 1. At least 6 or more independent biological samples were measured per category. **c** Bar plot shows transcriptional changes of target genes after *E*. *coli* injection in the brain at 6 hpi for control siblings and *foxq1a* and *foxq1b* mutants. *n* = 9–14 independent biological samples were measured per genotype. All error bars show standard error of means. Statistical significance in **b** and **c** was determined by multiple unpaired t-tests comparing between uninjected or sibling controls and the experimental groups with correction for multiple comparisons using the Sidak-Bonferroni method and without assuming equal variance. n.s., no statistical significance as defined by p > 0.05. *, p < 0.05; **, p < 0.01; ***, p < 0.001.

While *foxq1a* and *foxq1b* are not essential for innate immune cell development, they may affect the function of these cells, such as their ability to mediate an immune response. We developed a brain challenge assay (Fig 5A) to trigger innate immune activation by introducing live *E*. *coli* bacteria into the brain tectum of zebrafish larvae. We used this assay to address whether *foxq1a*, *foxq1b*, or both may regulate systemic response to *E*. *coli* challenge. We validated the effectiveness of our assay to trigger immune activation by showing that after brain challenge with bacteria or LPS at 6 hours post injection (hpi), we indeed found significant elevation of several innate immune activation genes systemically: interleukin 1 beta (*il1β*) [61,62,66], immune responsive gene (*irg1*) [67], neutrophil gene (*mpx*) [68], and tumor necrosis factor alpha (*tnfα*) [69] but not in water injected or uninjected controls (Fig 5B). We also tested whether *foxq1a* and *foxq1b* themselves could be transcriptionally regulated during a bacterial response to implicate a possible role in this process. Interestingly, we found that *foxq1a*, but not *foxq1b*, was significantly downregulated by about a factor of 2 in wildtype larvae after LPS or bacteria injection (Fig 5B).

To analyze the immune response of *foxq1* mutants, we employed the brain challenge assay on 4 dpf larval progenies of *foxq1a*^*bcz11/+*^ and *foxq1b*^*bcz18/+*^ single and double heterozygous incrosses (Fig 5C). We found that the expression levels of immune activation genes (*il1β*, *irg1*,*mpx*, *and tnfα*) in *foxq1* mutants at 6 hpi were not significantly different from WT siblings (Fig 5C). However, we cannot exclude the possibility that double *foxq1a* and *foxq1b* mutants may have deficient or aberrant transcriptional response to *E*. *coli*. Since *foxq1a* transcriptional level was downregulated in wildtype response to *E*. *coli* (Fig 5B), we also assessed whether this occurred normally in *foxq1* mutants as compared to WT siblings. qPCR data shows that *foxq1b* mutants had similar *foxq1a* expression as WT controls after bacterial exposure, but both heterozygous and homozygous *foxq1a* mutants had a modest but significant increase of ~50% in *foxq1a* RNA level (Fig 5C). The *foxq1a* upregulation was also found in *foxq1a* mutants at steady state, suggesting that the increase during the bacterial response may be due to an already higher baseline level rather than necessarily the bacterial exposure (S3 Fig). The results indicate that *foxq1a* and *foxq1b* may not be required for mediating an appropriate transcriptional response to *E*. *coli* challenge, but *foxq1a* may affect other aspects of this bacterial response as it is transcriptionally regulated.

## Discussion

Our study used zebrafish genetic mutants to investigate the possibility that FOXQ1 may have a role in innate immune cell development and function. Using our newly generated null mutations of the zebrafish homologs of human FOXQ1, *foxq1a* and *foxq1b*, we reveal that these genes are not required for the normal development and distribution of macrophages, neutrophils, and microglia. Furthermore, our data suggests that these two genes are not necessary for a stereotypical transcriptional response to *E*. *coli* exposure, albeit deficiency in both copies of *foxq1* was not assessed. It is possible, however, that *foxq1a* and *foxq1b* may have important roles in other innate immune functions such as during an infection or injury that were not examined in this study, which include cell migration and recruitment, phagocytosis, and host response to pathogens, viruses, fungi, and environmental toxins. Since *foxq1a* expression was found in macrophages and downregulated during a bacterial response, *foxq1a* may have a role in innate immunity that remains to be fully explored in zebrafish. Furthermore, we show that *foxq1a* is upregulated in the *foxq1a* mutants at steady state and during a bacterial response. This suggests that *foxq1a* may be forming a negative feedback loop in negatively regulating its own transcription.

The *foxq1a*^*bcz11*^ and *foxq1b*^*bcz18*^ mutants will provide useful genetic models to address previously unanswered questions regarding function of zebrafish *foxq1* in craniofacial development, possible gastrointestinal processes, and later adult organogenesis [51,52]. Consistent with the possibility that FOXQ1 has multiple roles in development, FOXQ1/Foxq1 is highly expressed in mouse stomach and kidney, and human gastric mucosa, bladder, salivary glands, trachea, intestines, and liver [2,16,70]. In addition, murine Foxq1 deletion mutants have deficiencies in gastric acid secretion and hair differentiation, and show partial penetrance for embryonic lethality [16,70]. The zebrafish *foxq1* mutants can also be invaluable for uncovering mechanisms mediating the role of FOXQ1 in cancer biology that remains poorly understood.

## Materials and methods

### Zebrafish lines and embryos

Embryos from wildtype (TL and AB), transgenic lines: *mpeg1*:*EGFP* [58,71] and *gata1*:*DsRed* [72], and heterozygous lines: *foxq1a*^*bcz11/+*^, *foxq1b*^*bcz18/+*^, and *foxq1a*^*bcz11/+*^ *foxq1b*^*bcz18/+*^ were raised at 28.5°C, and staged as described [73]. Homozygous mutants were derived from either single heterozygous incrosses or from double heterozygous incrosses. The latter yielded double *foxq1a*^*bcz11*^ *foxq1b*^*bcz18*^ mutants at a ratio of ~ 1/16. Embryos were treated with 0.003% 1-phentyl-2-thiourea (PTU) to inhibit pigmentation in sterilized zebrafish system water. This study was carried out in accordance with the animal protocol (16–160) approved by the Institute Animal Care and Use Committee at UNC Chapel Hill.

### Neutral red analysis

Microglia were scored in live larvae by neutral red vital dye staining assay as previously described [61,62]. In brief, 3 dpf larvae were stained with neutral red by immersion in system water supplemented with 2.5 μg/ml neutral red and 0.003% PTU at 28.5°C for 1 hour, followed by 1–2 water changes, and then analyzed 2–3 hours later using a stereomicroscope.

### Whole mount RNA *in situ* hybridization

In situ hybridization was performed using standard methods as described in [61,62,74]. Antisense riboprobes were transcribed from: *mfap4* plasmid previously cloned [61], *mpx* (full-length, Open Biosystems clone 6960294) [61], *foxq1a* plasmid (pCES150), which was made from a 877 bp fragment cloned from a wildtype zebrafish embryo cDNA library using primers F476: GCACTCATCATCTGCAACAGGTA and R1353: TGATATCCCGCGGTTGCAGG. Anti-sense *foxq1a* RNA probe was made from pCES150 digested with HindIII and transcribed by T7 RNA polymerase. To make the negative control RNA probe, *foxq1a* sense strand sequence was transcribed by T3 RNA polymerase using a PCR template made from the *foxq1a* plasmid pCES150. *foxq1b* anti-sense RNA probe was made from a PCR-based template encompassing 754 bp of coding sequence using forward primer: GTGTTGCGAGATCCCTCGCGTC and reverse primer: GTAAACACTGTGCAGTGGCGCGTC, where the reverse primer also included the T7 RNA polymerase recognition site.

### RNA extraction, RT-PCR and quantitative PCR (qPCR)

Total RNA was extracted from individual larvae using the RNAqueous-Micro Isolation Kit (Ambion). cDNA was made using oligo dT primer and SuperScript IV reverse transcriptase (Invitrogen). qPCR was performed on the Applied Biosystems QuantStudio Flex 6 Real-Time PCR System using probe-based assays or SYBR Green. The delta–delta ct method was used to determine the relative levels of mRNA expression between experimental samples and controls. *ef1a* was used as the reference gene for determination of relative expression of all target genes. Sequences of the qPCR probes and primer sets used in qPCR and RT-PCR analyses are listed in Table 1.

**Table 1:** Probes and primer sequences for qPCR and RT-PCR.

***ef1a***	probe	CTGGAGACAGCAAGAACGACCCAC	
*** ***	F primer	ACATCCGTCGTGGTAATGTG	
*** ***	R primer	TGATGACCTGAGCGTTGAAG	
***il1b***	probe	TCCGTCAAATGTCCCGGTTGGTTTA	
*** ***	F primer	ACCGGCAGCTCCATAAAC	
*** ***	R primer	GGTGTCTTTCCTGTCCATCTC	
***irg1***	probe	TTTAACACCGTGCTTCAGTGCAGC	
*** ***	F primer	ACTGGGAGTGGGTTTGATTG	
*** ***	R primer	GCATACTGAGGTGGAAGAGATG	
***mpx***	probe	TATAGCATGGTCACGCCCTCTTTGC	
	F primer	TGCCTTCACATCCCACATAG	
	R primer	GGAGCAGACAATCCACAGAA	
***tnfa***	F primer	GCGCTTTTCTGAATCCTACG	
	R primer	TGCCCAGTCTGTCTCCTTCT	
***foxq1a***	probe	TAAGGGATCCTTCGAGACCGTGGG	
	F primer	GTGCGCCATAATCTGTCTCTAA	
	R primer	CGGGTTCAGCATCCAGTAAT	
***foxq1b***	probe	ATGGGATTTCCGCCACAGAGCA	
	F primer	TCCTCACTTTCTGCCAGTTG	
	R primer	ACATTCGACACCTCAGAACTG	
***DsRed***	F primer	TCCGAGGACGTCATCAAGGAGTTC	sorted cells
** **	R primer	GGCGGGGTGCTTCACGTACAC	sorted cells
***foxq1a***	foxq1a_F531	GCAGAAGGAGAAAGCGCATTAG	sorted cells
** **	foxq1a_R773	AGGATCGAAGCATGACATACGG	sorted cells
***foxq1b***	foxq1b_F540	CAGCGAGTACACTTTTGCAGAC	sorted cells
** **	foxq1b_R839	AATCTGCTCTGTGGCGGATAG	sorted cells
***GFP***	F primer	TATATCATGGCCGACAAGCA	sorted cells
** **	R primer	CTGGGTGGCTCAGGTAGTGG	sorted cells
***hbbe1*.*1***	hbbe1.1_F139	GCTCTGGCAAGGTGTCTCATC	sorted cells
** **	hbbe1.1_R465	AGCGATGAATTTCTGGAAAGCG	sorted cells
***irf8***	irf8-218-F	CAGCGACATGGAAGACCAGATTG	sorted cells
** **	irf8-647-R	GTGGTCACCATGTTGTCCACCATC	sorted cells
***mpeg1***	mpeg1_F1	ACAACACCACCTTGTTACACTCT	sorted cells
** **	mpeg1_R1	ACAACTGCTGGATTTGGTCAATG	sorted cells
***foxq1a***	foxq1a_F476	TGATATCCCGCGGTTGCAGG	2dpf/8dpf/gut
** **	foxq1a_R1353	GCACTCATCATCTGCAACAGGTA	2dpf/8dpf/gut
***foxq1b***	foxq1b_F159	ATGGATGTGAACGTGGCTTCA	2dpf/8dpf/gut
** **	foxq1b_R961	GGGTGAAATCCAGCCTCTTGTA	2dpf/8dpf/gut

### CRISPR-Cas9 targeted mutagenesis of *foxq1a* and *foxq1b*

The target genes were *foxq1a* (NCBI accession: NM_001243344.1; Gene ID: 100537750) and *foxq1b* (NCBI accession: NM_212907.1; Gene ID: 405843). Co-injection of Cas9 mRNA and guide RNAs (gRNAs) was conducted in wildtype 1-cell stage zebrafish embryos. Cas9 mRNA was transcribed from XbaI linearalized pT3TS-nCas9n plasmid (Addgene #46757) [53] using mMessage mMachine T3 Kit (Ambion) according to the manufacturer’s instructions. CRISPR targets for gRNA designs were identified using CHOPCHOP (http://chopchop.cbu.uib.no) [54]. The following gene-specific oligonucleotides using T7 promoter were used to make gRNAs as previously described [54]; gene-specific target sequences are underlined: *foxq1a* gRNA-1 5’-TAATACGACTCACTATAGGGTTCGAAGAGTGACAGGGGTTTTAGAGCTAGAAATAGCAAG-3’, *foxq1a* gRNA-2 5’-TAATACGACTCACTATAGGACTACGACTCCAAGCCTGGTTTTAGAGCTAGAAATAGCAAG-3’, *foxq1a* gRNA-3 5’- TAATACGACTCACTATAGGAGTTGTGCAGCGATGCTGGTTTTAGAGCTAGAAATAGCAAG-3’, *foxq1b* gRNA-1 5’-TAATACGACTCACTATAGGCTTCACCGCTGTCCACGGGTTTTAGAGCTAGAAATAGCAAG-3’, *foxq1b* gRNA-2 5’-TAATACGACTCACTATAGGTTCCTCCTCCGTGGACAGGTTTTAGAGCTAGAAATAGCAAG-3’, *foxq1b* gRNA-3 5’-TAATACGACTCACTATAGGAGCCCAATTCCTCCTCCGGTTTTAGAGCTAGAAATAGCAAG-3’. In vitro transcription of gRNAs from assembled oligonucleotides was conducted using the HiScribe T7 Quick High Yield RNA Synthesis Kit (NEB). Injected clutches of embryos validated to contain CRISPR mediated mutagenesis by a T7 endonuclease assay were raised as F0 fish. Incross and outcross of F0 adults produced F1 progenies, which were genotyped and sequenced at adult stages to identify F1 founders carrying mutations of interest. These founders were isolated and outcrossed to raise new mutant lines, which yielded the *foxq1a*^*bcz11/+*^ and *foxq1b*^*bcz18/+*^ heterozygous fish used in this study.

### Cell dissociation and fluorescence activated cell sorting (FACS)

Dissociation of pools of 25–50 2.5 dpf transgenic zebrafish embryos either carrying the macrophage reporter (*mpeg1*:*EGFP*) or the erythrocyte reporter (*gata1*:*DsRed*) was conducted using a protocol adapted from [75]. Fluorescently tagged cells were sorted from non-fluorescent cells using the BD FACSAria cell sorter equipped with three lasers. Non-fluorescent control embryos were used for setting gates in FACS for GFP and DsRed positive cells. BD FACSDiva V8.0.1 software was used for cell sorting and FACS plots. Total RNA was extracted from the different populations of cells (GFP positive, GFP negative, DsRed positive, DsRed negative) using the RNAqueous-Micro Isolation Kit (Ambion), and made into cDNA for RT-PCR analyses as described above.

### Genotyping bcz11 and bcz18 alleles

To genotype *foxq1a*^*bcz11*^, the following primers were used: *foxq1a F* 5’-CGGTGTTTTTGTGACTTGATTT-3’ and *foxq1a R* 5’-AAGAGTATGGAGGTTTGGGTCTC-3’ in a PCR assay that yielded differentially sized products to distinguish the mutant allele which has a 95 bp deletion compared with the wildtype allele. The expected sizes for mutant band is 240 bp and wildtype band is 335 bp. To genotype *foxq1b*^*bcz18*^, a PCR and restriction enzyme combined assay was used. Primers *foxq1b F* 5’-AAGCAACTCATCTGACCTGACA-3’ and *foxq1b R* 5’-GGGTCTACGCGTATATGGTTTC-3’ were used to yield PCR products of 217 bp, followed by a digest with BsaJI, which only recognizes the wildtype sequence.

### Immune challenge

Bacteria were prepared from 3 mL overnight cultures, each derived from a single *Escherichia coli* colony, which were centrifuged and re-suspended in 500 μL of PBS. The 500 μL bacteria mix (~1.6 x 10^6^ cfu/μL) was supplemented with 1 μL of 5 mg/ml AlexaFluor 568 or AlexaFluor 488 conjugated dextran (10 kDa) from Invitrogen. Other reagents used for brain injections were: ultra-pure water supplemented with 0.05 mg/ml fluorescently labeled dextran (10 kDa) for the control water samples, and lipopolysaccharides (LPS) derived from 0111:B4 *E*. *coli* (L3024 Sigma) at 4.5 mg/ml was supplemented with 0.05 mg/ml fluorescently labeled dextran (10 kDa). For all injections, 1 nL of the reagent mix was injected into the right brain tectum of 4 dpf larvae, and the fluorescent dextran was used to validate the injection site. Larvae that were successfully injected were collected 6 hours post injection (hpi) and immediately processed for total RNA lysis. RNA extraction and subsequent steps for qPCR analyses are described above. All collected larvae derived from heterozygous intercrosses were genotyped after RNA extraction.

## Supporting information

S1 Fig RT-PCR and Sanger sequencing analyses of the mutations *bcz11* and *bcz18* confirm the expression of the expected mutant transcripts and show no alternative splicing. Primers used to amplify the approximate full-length coding region of each gene were: start-F 5’-GCACTCATCATCTGCAACAGGTA and end-R 5’-TGATATCCCGCGGTTGCAGG for *foxq1a* (~900 bp); and start-F 5’-AAGCAACTCATCTGACCTGACA and end-R 5’-GTAAACACTGTGCAGTGGCGCGTC for *foxq1b* (~1150 bp). (a) Inverted DNA gel image of the RT-PCR analysis of *foxq1a* mRNA shows the expected ~900 bp transcript in WT siblings and a single mutant transcript with a 95 bp deletion in homozygous *bcz11* mutants, indicating no alternative splicing in the mutants. Gel image shows 2 independent samples per genotype. (b) Sanger sequencing analysis of the *foxq1a* transcript from four *bcz11* mutants shows the expected 95 bp deletion that caused a frameshift leading to an early stop codon at the beginning of the gene. (c) RT-PCR analysis of the *foxq1b* transcript in WT and *bcz18* mutants shows the expected product size of ~1150 bp and no apparent splice variants. Gel image shows 2 independent samples per genotype. (d) Sanger sequencing analysis of the *foxq1b* transcript from four *bcz18* mutants confirms the indel mutation that leads to a frameshift and early stop codon. aa, amino acid; sib, sibling; Mut, mutant.
(TIF)

S2 Fig RNA expression pattern of *foxq1a* and *foxq1b* in zebrafish embryos. **a-d** *foxq1a* gene expression. **a** Expression in ventral head region is found in 1.5 dpf embryo (arrow). **b** Prominent expression at 2.5 dpf in the craniofacial region (arrows, higher magnification in **c**), on the yolk (arrows, higher magnification in **d**), and along the body abutting the yolk. Yolk expression is not found in all embryos analyzed whereas the craniofacial expression is. **e-f** As a negative control, whole mount in situ hybridization using the sense RNA probe for *foxq1a* does not show any expression at all stages analyzed from 1–3 dpf. **g** *foxq1b* expression is prominent in the craniofacial jaw region. Left, lateral view (arrow). Right, ventral view (arrows).
(TIF)

S3 Fig *foxq1a* is transcriptionally upregulated in *foxq1a* mutants. A modest upregulation of *foxq1a* RNA expression at ~ 44% on average is found in *foxq1a* mutants at 4 dpf at steady state in the absence of any immune challenge. *n* = 6 independent biological samples were measured per genotype. All error bars show standard error of means.
(TIF)
