# Supplementary figures and images for: Genetic analysis of zebrafish homologs of human FOXQ1, foxq1a and foxq1b, in innate immune cell development and bacterial host response
Source: PLoS One. 2018 Mar 13;13(3):e0194207. doi: 10.1371/journal.pone.0194207 (PMC5849333; doi:10.1371/journal.pone.0194207)

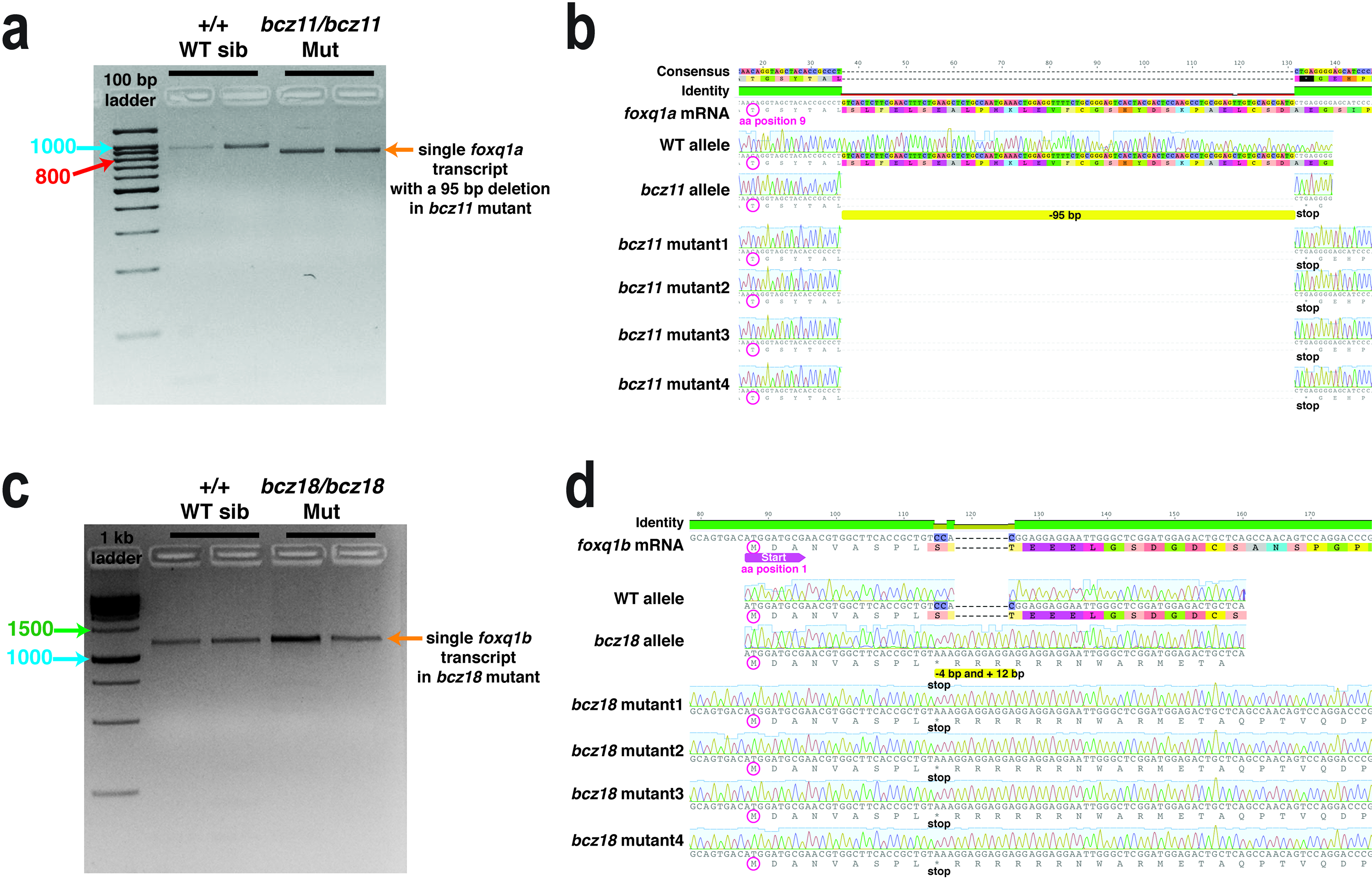

Supplement: S1 Fig — Primers used to amplify the approximate full-length coding region of each gene were: start-F 5’-GCACTCATCATCTGCAACAGGTA and end-R 5’-TGATATCCCGCGGTTGCAGG for foxq1a (~900 bp); and start-F 5’-AAGCAACTCATCTGACCTGACA and end-R 5’-GTAAACACTGTGCAGTGGCGCGTC for foxq1b (~1150 bp). (a) Inverted DNA gel image of the RT-PCR analysis of foxq1a mRNA shows the expected ~900 bp transcript in WT siblings and a single mutant transcript with a 95 bp deletion in homozygous bcz11 mutants, indicating no alternative splicing in the mutants. Gel image shows 2 independent samples per genotype. (b) Sanger sequencing analysis of the foxq1a transcript from four bcz11 mutants shows the expected 95 bp deletion that caused a frameshift leading to an early stop codon at the beginning of the gene. (c) RT-PCR analysis of the foxq1b transcript in WT and bcz18 mutants shows the expected product size of ~1150 bp and no apparent splice variants. Gel image shows 2 independent samples per genotype. (d) Sanger sequencing analysis of the foxq1b transcript from four bcz18 mutants confirms the indel mutation that leads to a frameshift and early stop codon. aa, amino acid; sib, sibling; Mut, mutant. (TIF) [file pone.0194207.s001.tif]

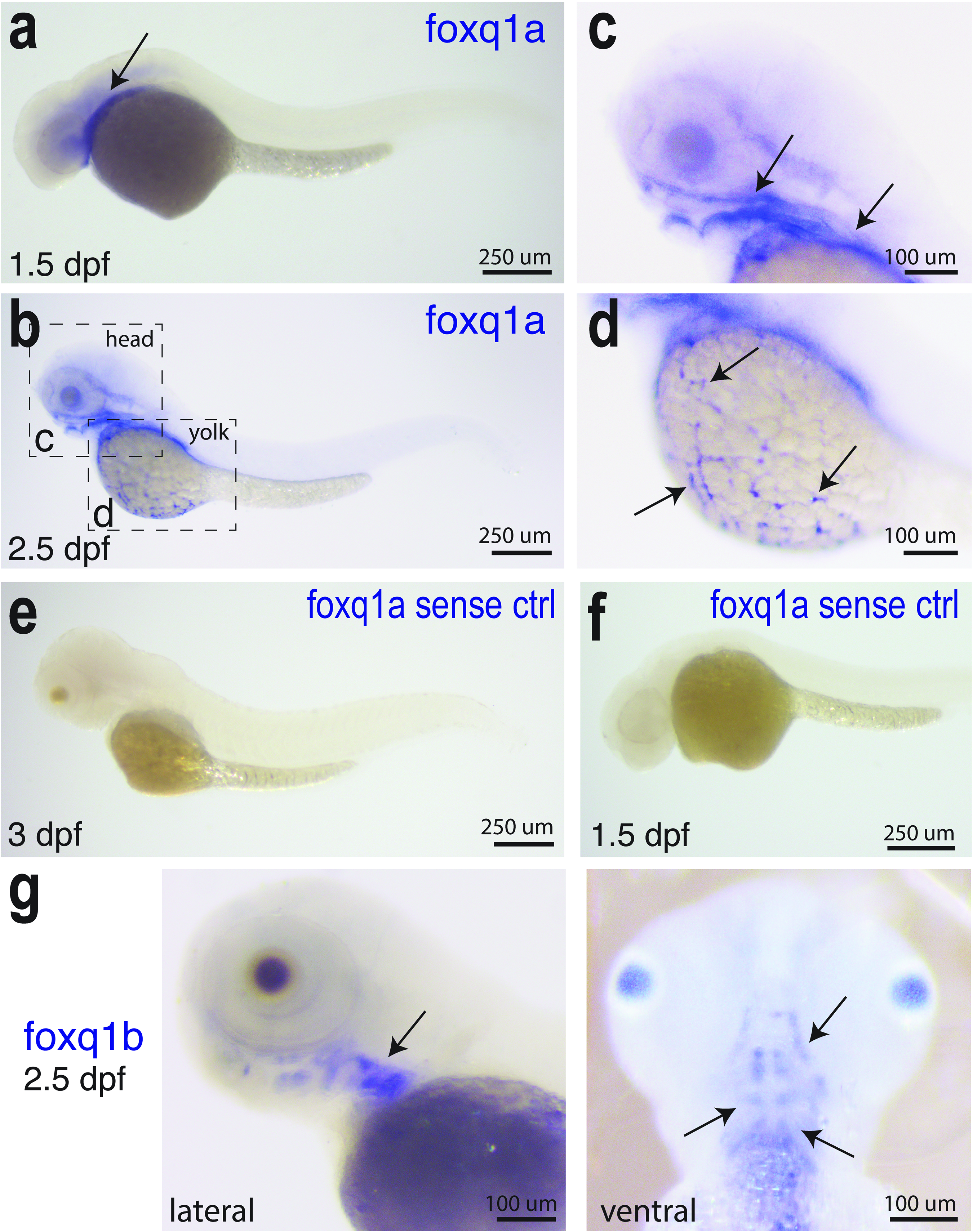

Supplement: S2 Fig — a-d foxq1a gene expression. a Expression in ventral head region is found in 1.5 dpf embryo (arrow). b Prominent expression at 2.5 dpf in the craniofacial region (arrows, higher magnification in c), on the yolk (arrows, higher magnification in d), and along the body abutting the yolk. Yolk expression is not found in all embryos analyzed whereas the craniofacial expression is. e-f As a negative control, whole mount in situ hybridization using the sense RNA probe for foxq1a does not show any expression at all stages analyzed from 1–3 dpf. g foxq1b expression is prominent in the craniofacial jaw region. Left, lateral view (arrow). Right, ventral view (arrows). (TIF) [file pone.0194207.s002.tif]

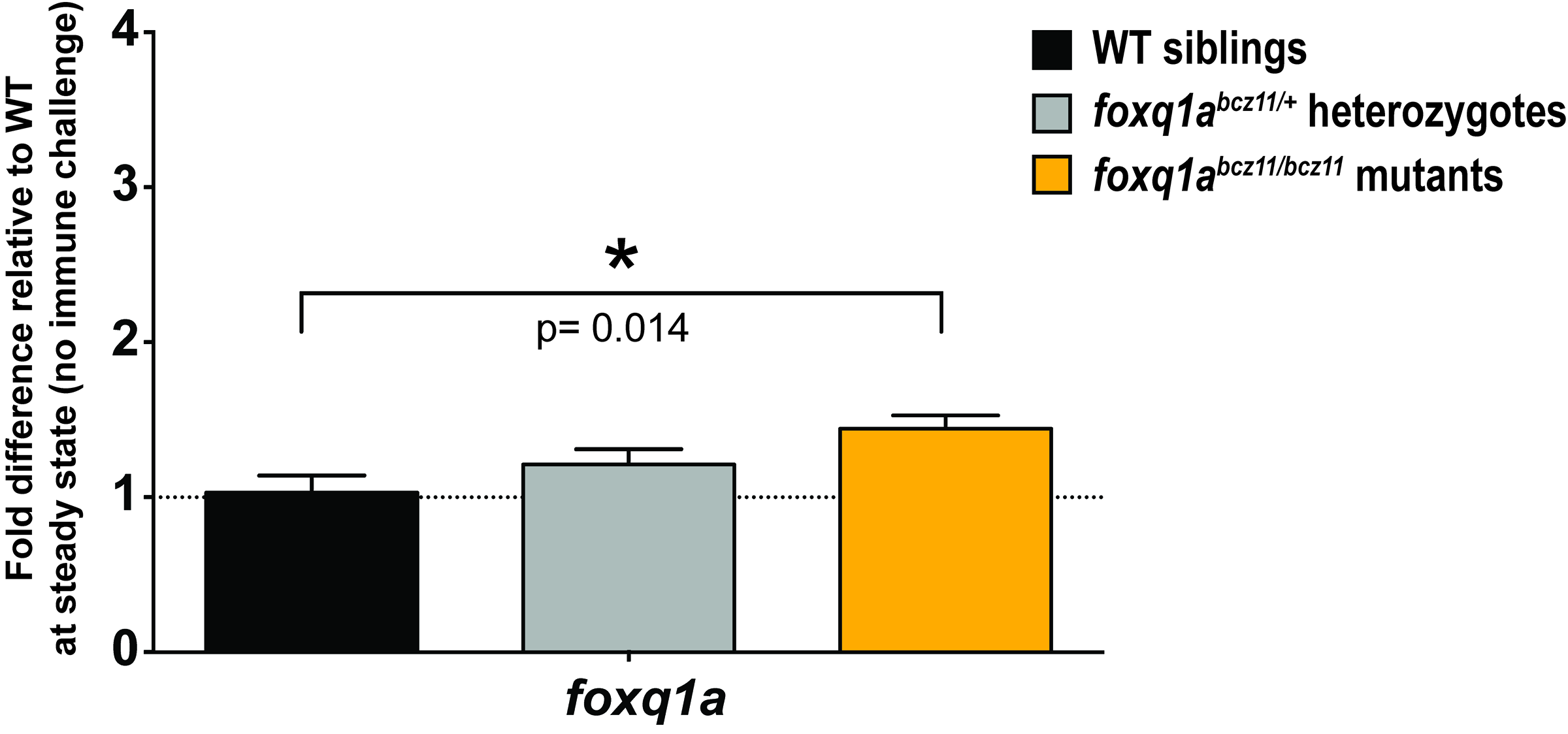

Supplement: S3 Fig — A modest upregulation of foxq1a RNA expression at ~ 44% on average is found in foxq1a mutants at 4 dpf at steady state in the absence of any immune challenge. n = 6 independent biological samples were measured per genotype. All error bars show standard error of means. (TIF) [file pone.0194207.s003.tif]
